# The Differentiation of Amnestic Type MCI from the Non-Amnestic Types by Structural MRI

**DOI:** 10.3389/fnagi.2016.00052

**Published:** 2016-03-30

**Authors:** Gábor Csukly, Enikő Sirály, Zsuzsanna Fodor, András Horváth, Pál Salacz, Zoltán Hidasi, Éva Csibri, Gábor Rudas, Ádám Szabó

**Affiliations:** ^1^Department of Psychiatry and Psychotherapy, Semmelweis UniversityBudapest, Hungary; ^2^Department of Neurology, National Institute of Clinical NeurosciencesBudapest, Hungary; ^3^Department of Neurology, Hospital at Péterfy Sándor StreetBudapest, Hungary; ^4^Magnetic Resonance Imaging Research Center, Semmelweis UniversityBudapest, Hungary

**Keywords:** mild cognitive impairment, amnestic, non-amnestic, MRI, neuropsychological test

## Abstract

**Introduction:** While amnestic mild cognitive impairment (aMCI) and non-amnestic mild cognitive impairment (naMCI) are theoretically different entities, only a few investigations studied the structural brain differences between these subtypes of mild cognitive impairment. The aim of the study was to find the structural differences between aMCI and naMCI, and to replicate previous findings on the differentiation between aMCI and healthy controls.

**Methods:** Altogether 62 aMCI, naMCI, and healthy control subjects were included into the study based on the Petersen criteria. All patients underwent a routine brain MR examination, and a detailed neuropsychological examination.

**Results:** The sizes of the hippocampus, the entorhinal cortex and the amygdala were decreased in aMCI relative to naMCI and to controls. Furthermore the cortical thickness of the entorhinal cortex, the fusiform gyrus, the precuneus and the isthmus of the cingulate gyrus were significantly decreased in aMCI relative to naMCI and healthy controls. The largest differences relative to controls were detected for the volume of the hippocampus (18% decrease vs. controls) and the cortical thickness (20% decrease vs. controls) of the entorhinal cortex: 1.6 and 1.4 in terms of Cohen's d. Only the volume of the precuneus were decreased in the naMCI group (5% decrease) compared to the control subjects: 0.9 in terms of Cohen's d. Significant between group differences were also found in the neuropsychological test results: a decreased anterograde, retrograde memory, and category fluency performance was detected in the aMCI group relative to controls and naMCI subjects. Subjects with naMCI showed decreased letter fluency relative to controls, while both MCI groups showed decreased executive functioning relative to controls as measured by the Trail Making test part B. Memory performance in the aMCI group and in the entire sample correlated with the thickness of the entorhinal cortex and with the volume of the amygdala.

**Conclusion:** The amnestic mild cognitive impairment/non-amnestic mild cognitive impairment separation is not only theoretical but backed by structural imaging methods and neuropsychological tests. A better knowledge of the MCI subtypes can help to predict the direction of progression and create targeted prevention.

## Introduction

Major neurocognitive disorders (NCD) or dementia including Alzheimer's disease (AD) are a devastating problem of our aging society having tremendous social, economic and medical burden. Since we do not have effective treatment for NCD at the moment, recent research have focused on the detection of early symptoms. Converging evidence from many recent studies revealed that pathologic process of AD starts decades prior to the first symptoms of cognitive decline (Mattsson et al., 2009). The intermediate stage between the expected cognitive decline in physiological aging and the severe decline in dementia known as “mild cognitive impairment” (MCI) has gained a lot of interest. “In MCI mild impairment of cognitive skills can be revealed by neuropsychological tests, while global cognitive functions and everyday activities are preserved” (Petersen, 2004). The higher conversion rate to NCD in MCI gives the clinical significance of this pre-disease condition. Conversion rate to NCD is 10–15% annually in MCI compared to the annual rate of 1–4% in the average elderly population (Petersen et al., 2001; Bischkopf et al., 2002). The majority of these patients develop clinical AD. It should be noted that MCI could be also a stable or reverse condition not progressing into dementia. Longitudinal epidemiologic studies reported variant conversion rate and a relatively frequent reversion to normal cognition (Larrieu et al., 2002; Ganguli et al., 2004). Different findings suggest instability in the diagnostic accuracy and a need for more specific identification between the MCI patients (Han et al., 2012). In view of the above it is understandable that several studies target the symptoms and differences from the average population that are closely linked to the development of dementia and can therefore be used to assist the early diagnosis.

Patients with MCI can be categorized further as amnestic (aMCI) and non-amnestic MCI (naMCI). In aMCI the memory loss is predominant and it is associated with high risk to further conversion to AD (Grundman, 2004). Individuals with naMCI have impairments in other domains than memory and have a higher risk to convert to other dementia forms such as diffuse Lewy body dementia. Both types can be categorized further to single-domain and multi domain subtypes, however in the present investigation no further categorization was done due to the limited sample size. Significant types of biomarkers of MCI have been tested with the aim to identify the special features of patients conversing into AD. While results of cerebrospinal fluid biomarkers (Hansson et al., 2006) and positron emission tomography studies (Mosconi et al., 2004) pointed out highly variant specificity and sensitivity, structural magnetic resonance imaging studies revealed impressive results (Jack et al., 1999; deToledo-Morrell et al., 2004). Interestingly, several previous studies investigated the differences between subjects with MCI and healthy controls, only a few studies tried to differentiate between the MCI subtypes (Zhang et al., 2011; Serra et al., 2013).

While aMCI and naMCI are theoretically different entities, only a few investigations studied the structural brain differences between these subtype of MCI (Serra et al., 2013). The aim of the study was to find the structural differences between aMCI and naMCI, and to replicate previous findings on the differentiation between aMCI and healthy controls. Based on previous studies on MCI and conversion to dementia we focused on structures of the temporal lobe and the neighboring regions (Chiang et al., 2011): the entorhinal cortex, the hippocampus, the parahippocampal cortex, the amygdala, the fusiform gyrus, the precuneus, the posterior cingulate cortex, and the isthmus of cingulate gyrus.

## Methods

This study is a continuation of our previously published work (Sirály et al., 2015). Since a similar neuropsychological test battery, and the same MRI acquisition and processing pipeline has been used in the present study, methods and procedures have been described similarly in both papers.

### Ethics statement

The experiments were conducted in full compliance with the Helsinki Declaration and all relevant national and international ethical guidelines. The research was approved by the National Ethics Committee, Budapest, Hungary. All procedures were carried out only after written informed consent was obtained from the participants. All potential participants who declined to participate or otherwise did not participate were not disadvantaged in any way by not participating in the study.

### Participants

The study was carried out in the Department of Psychiatry and Psychotherapy, Semmelweis University, Budapest. Altogether 62 subjects were enrolled in the study. All subjects applied to participate in a cognitive training program announced among general practitioners and in a Retirement Home (The study is registered at ClinicalTrials.gov, identifier is “NCT02310620”). All subjects were able to lead independent lives. Basic demographic and neuropsychological data are summarized in Table 1.

**Table 1:** **Demographic data and result of basic neuropsychological tests**.

	**Control (*n* = 24)**	**naMCI (*n* = 18)**	**aMCI (*n* = 20)**	***p-value***
Age	65.4 (7.6)	70.9 (7.3)	70.9 (11.3)	n.s.^*^
Education^a^	4%/38%/58%	11%/28%/61%	15%/25%/60%	n.s.^*^
Gender (Female)	71%	61%	60%	n.s.^*^
Rey Auditory Verbal Learning Test 1–5 sum^b^	53.2 (8.1)	47.1 (9.9)	29.6 (7.3)	*p* < 0.0001
ACE Total Score^c^	94.0 (3.3)	89.1 (5.1)	82.2 (7.4)	*p* < 0.0001
ACE VL/OM-ratio^d^	2.6 (0.4)	2.5 (0.4)	3.1 (0.8)	*p* = 0.003
Mini mental state examination total score^e^	28.5 (1.4)	28.3 (1.0)	27.5 (1.7)	n.s.^*^
Geriatric depression scale score^f^	3.2 (2.7)	5.0 (2.8)	4.1 (3.2)	n.s.^*^
STAI score^g^	37.3 (10.4)	37.1 (9.3)	35.9 (8.4)	n.s.^*^

*aMCI, Amnestic mild cognitive impairment; naMCI, non-amnestic mild cognitive impairment, ACE, Addenbrooke's Cognitive Examination; STAI, state-trait anxiety inventory*.

a *Participants were categorized into three education groups: 1, ≤ 12 years; 2, high school graduation (12 years education); 3, more than 12 years education*.

b *Sum of all words in the first five trials. The maximum score is 75. Normative data can be found in Table 2A*.

c *The maximum score is 100*.

d *VL/OM: verbal fluency and language points/orientation and delayed recall ratio can be defined based on ACE. Result below 2,2 indicate frontotemporal dementia and result over 3,2 indicate Alzheimer's disease*.

e *The maximum score is 30. Normative data can be found in the Table 2B*.

f *The maximum score is 15*.

g *State-Trait Anxiety Inventory. The maximum score is 80*.

* *n.s. (not significant) = p > 0.05*.

Subjects with aMCI and naMCI, and healthy controls were included into the study based on the Petersen criteria (Petersen, 2004). The Petersen criteria include subjective memory complaint corroborated by an informant together with preserved everyday activities, a memory impairment based on a standard neuropsychological test, preserved global cognitive functions and finally the exclusion of dementia. It does not specify a neuropsychological test for the assessment of memory impairments, therefore we applied the Rey Auditory Verbal Learning Test (RAVLT), which is the most frequently used test based on the literature (Gomar et al., 2011). For the differentiation between aMCI and healthy controls we applied a cutoff score of 1 SD under population mean standardized for age and gender. Participants, who scored under the cutoff value either in the delayed recall subscore or in the total score, was put into the aMCI group. The applied criteria are based on the recommendations of the National Institute on Aging—Alzheimer's Association workgroups on diagnostic guidelines for Alzheimer's disease (Albert et al., 2011). The exact cutoff scores for the RAVLT for the different age groups are provided in Table 2A. Subjects who were not in the aMCI group, but scored 1 SD under the population mean standardized for age and gender/education either in the Trail making Test B or in the Addenbrooke's Cognitive Examination (ACE), were put in the naMCI group. An additional criterion for the naMCI group was a lower than 3.2 VLOM (verbal fluency + language score/orientation + memory score) ratio in the ACE to exclude possible aMCI subjects from the naMCI group (these subjects were excluded from the study).

**Table 2:** **(A). Rey Auditory Verbal Learning Test (RAVLT): normative data and cut-off scores for Mild Cognitive Impairment (MCI) adjusted for age; (B) Mini Mental Examination Test (MMSE): cut-off scores for dementia adjusted for age and education**.

**(A)**									
	**Age groups**	**50–59**		**60–69**		**70**+			
Total score (sum of trials 1–5)^a^	Mean (SD)	47.6 (8.1)		43.4 (7.7)		37.1 (7.5)			
	Cutoff score	39		35		29			
Delayed Recall^b^	Mean (SD)	9.9 (3.2)		8.8 (3.0)		7.0 (2.4)			
	Cutoff score	6		5		4			
**(B)**									
**Education Groups**	**Age groups**	**50–54**	**55–59**	**60–64**	**65–69**	**70–74**	**75–79**	**80–84**	**85** +
5–8 years	Cutoff score	23	23	23	23	23	21	21	17
9–12 years or high school diploma	Cutoff score	25	25	25	25	24	24	21	21
College experience or higher degree	Cutoff score	27	27	27	27	25	25	25	24

a *The summarized number of learned words in the first five trial. The maximum score in this subtest is 75*.

b *The number of recalled words after 20–30 min. The maximum score on this subtest is 15*.

*SD: standard deviation*.

Subjects with dementia were excluded from the study according to the Mini Mental State Examination (MMSE) scores standardized for age and education (Strauss et al., 2006b). The exact cutoff scores for the MMSE in the different age and education groups are provided in Table 2B. Subjects with history of head trauma, epilepsy or stroke, or diagnosis of acute psychiatric disorder, schizophrenia or mania, alcohol dependency were also excluded from the study.

### MR image acquisition and processing

All subjects were examined by brain MRI producing high resolution structural images, which were used for further processing and analysis. Image acquisitions were done at the MR Research Center, Semmelweis University, Budapest on a 3 Tesla Philips Achieva clinical MRI scanner equipped with an eight-channel SENSE head-coil. The high resolution, whole brain anatomical images were obtained using a T1 weighted 3 dimensional spoiled gradient echo (T1W 3D TFE) sequence. 180 contiguous slices were acquired from each subject with the following imaging parameters: TR = 9.7 ms; TE = 4.6 ms; flip angle = 8°; FOV of 240 × 240 mm; voxel size of 1.0 × 1.0 × 1.0 mm.

Cortical reconstruction and volumetric segmentation were performed by Freesurfer 5.3 image analysis suite, which is documented and freely available for download online (http://surfer.nmr.mgh.harvard.edu/). The technical details of these procedures are described in prior publications, we made no changes to this pipeline. Briefly, image processing includes motion correction (Reuter et al., 2010), removal of non-brain tissue using a hybrid watershed/surface deformation procedure (Segonne et al., 2004), automated Talairach transformation, segmentation of the subcortical white matter, and deep gray matter volumetric structures (including hippocampus, amygdala, caudate, putamen, ventricles) (Fischl et al., 2004) intensity normalization, tessellation of the gray matter white matter boundary, automated topology correction, and surface deformation following intensity gradients to optimally place the gray/white and gray/cerebrospinal fluid borders at the location where the greatest shift in intensity defines the transition to the other tissue class (Dale et al., 1999; Fischl and Dale, 2000). Once the cortical models were completed, Freesurfer performed a number of deformable procedures for in further data processing and analysis. Steps included surface inflation (Fischl et al., 1999a), registration to a spherical atlas which utilized individual cortical folding patterns to match cortical geometry across subjects (Fischl et al., 1999b), parcellation of the cerebral cortex into units based on gyral and sulcal structure (Fischl et al., 2004), and creation of a variety of surface based data including maps of curvature and sulcal depth. Finally cortical models and the results of segmentation were quality checked and manually corrected on each subject, however correction showed no significant changes to the results.

### Procedures

The neuropsychological examinations were carried out on weekdays between 8 a.m. and 4 p.m. The examinations consisted of computerized (i.e., PAL test) and paper based neuropsychological tests. The tests took place in a separate well-lit room where only the patient and an examiner were present. Reference tests were also completed and evaluated according to the recommendations of the Neuropsychological Compendium (Strauss et al., 2006a), while the computerized tests were evaluated by the software.

Neuropsychological tests were administered by two previously trained medical students under the supervision of a psychologist and a psychiatrist. During the assessment of the tests the guidelines of the Neuropsychological Compendium (Strauss et al., 2006a) were followed. The paper based tests were evaluated by the same psychologist and a psychiatrist according to the compendium. The Paired Associates Learning Test (PAL test) were evaluated automatically by a software. MR data were also analyzed and evaluated automatically by the Freesurfer and SAS software; therefore no subjective judgments were involved in the analysis of neuroimaging data. Since the assessment and evaluation of the PAL test, and neuroimaging data were totally automatic, the bias from human judgments were low (limited only to the evaluation of the paper based tests).

Subjects with dementia were excluded from the study based on the Mini Mental State Examination (MMSE). The MMSE is a standard test; its effectiveness was proven by several studies, as a useful method in differentiating between subjects with dementia and healthy controls (Petersen et al., 1999; Gomar et al., 2011). The majority of the previous studies used the cut off score of 26 or the age adjusted score for dementia. The subtasks of the test assess orientation, central executive function, rapid association formation, verbal identification ability, and the ability to analyze and synthesize.

The Addenbrooke's Cognitive Examination was used to assess the global cognitive performance, including orientation, attention, memory, verbal fluency, verbal, and visuospatial skills (Mathuranath et al., 2000; Alexopoulos et al., 2010).

The Rey Auditory Verbal Learning Test was used for the detailed assessment of memory functions based on Petersen criteria. Rey test evaluates verbal learning and memory (Rey, 1958). A list of 15 words (list A) should be repeated by the subject immediately. This test is repeated 5 times. Then another list of 15 words (list B or interference list) is presented once that should be recalled. Then list A should be recalled without repeating, and then this task is repeated after 30 min.

The Trail Making test, Part A and Part B (number connection; REITAN, 1955; Tombaugh, 2004; Alexopoulos et al., 2010) is used to evaluate selective attention, executive functions, and cognitive flexibility. In Part A, randomly distributed numbers should be connected in numerical order, while in Part B randomly distributed numbers and letters are displayed. The subject is instructed to connect the numbers and letters in a pre-defined order. The dependent variable is the time required to complete the test. Part A of TMT measures executive functions and attention, while in the performance on Part B cognitive flexibility is also reflected.

The results of the neuropsychological tests are summarized in Table 1.

All participants completed a form in which they evaluated their own health condition and memory function; furthermore, they had to report on their computer and internet use, recreational activities, alcohol consumption, dietary habits, and smoking (Yesavage, 1988). The Geriatric Depression Scale (GDS) was used to assess depressive symptoms, while anxiety symptoms were measured by the Spielberger State-Trait Anxiety Inventory (STAI; Spielberger et al., 1970).

Visuospatial memory was measured by an implementation of the PAL test used in several neuropsychological test batteries (Siraly et al., 2013). “In the PAL test windows open up in random positions on the screen after each other for 3 s with abstract shapes shown in one or more windows. Other windows remain blank depending on the difficulty level. When all squares were shown, the previously shown shapes appear in the center of the screen and the participants have to decide in which window they saw that shape before. The test consist of five different levels in eight stages in total, the number of shapes increases from 1 to 8 on the different stages. The subjects had 10 trials to complete a given stage, otherwise the test ended. The arrangement of windows was asymmetrical in the test and it changed from stage to stage.” (Siraly et al., 2013) During the computerized test subjects were seated comfortably at a distance of half a meter from the computer screen and following prior information they solved the tasks with the use of a mouse.

### Statistical analysis

Differences between study groups in brain volumes, cortical thickness and cognitive performance were analyzed by General Linear Model Analysis (GLM in SAS 9.2) with age and gender as covariates. Volumes were standardized for total intracranial volume (TIV), and are given in percentage. In order to analyze between group differences post hoc t comparisons were applied, where *p*-values were adjusted to 0.05/3 = 0.016, where three stands for the number of between group comparisons.

The relationship between the results of the neuropsychological tests and the size of the CNS structures were analyzed by Pearson correlations. Correlations with the number of stages completed in the PAL test were analyzed by Spearman Correlation, since the distribution of this variable deviated largely from the normal distribution.

## Results

### Differences in cortical volumes, cortical thickness and cognitive performance between subjects with aMCI, nAMCI, and controls

**Figure 1:**
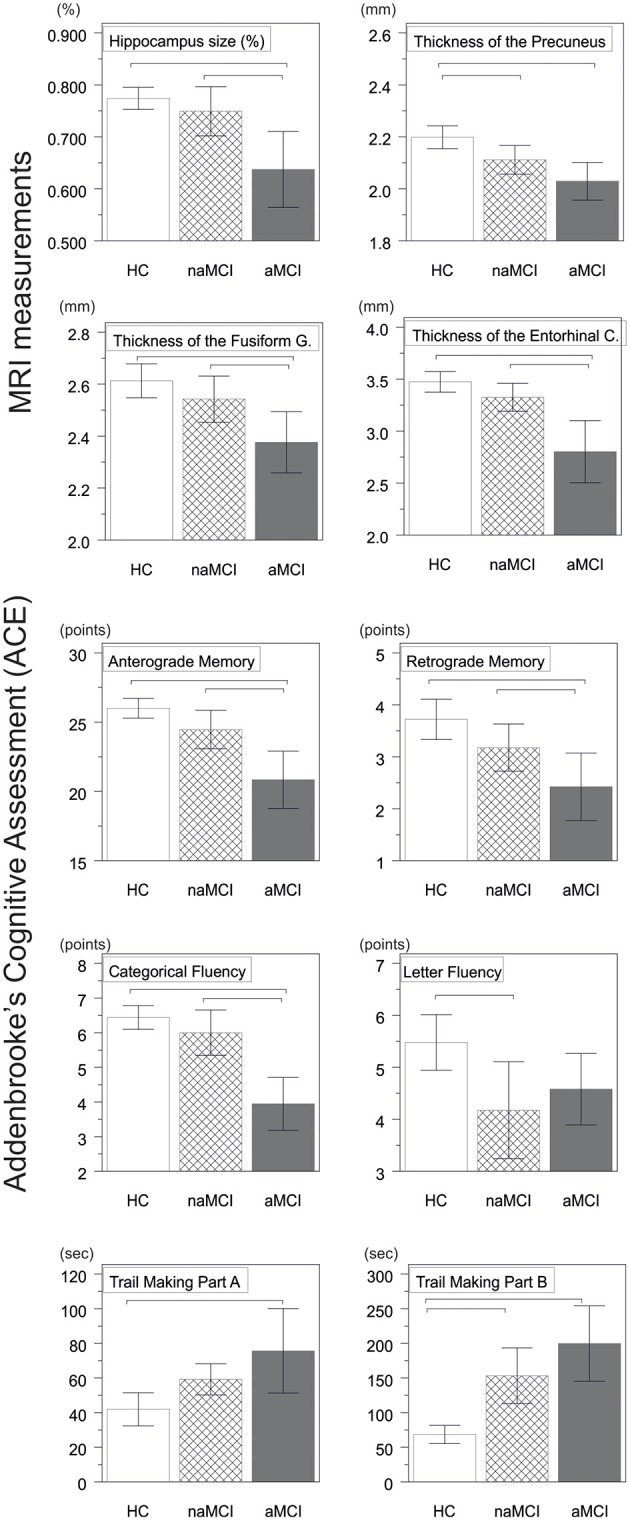
**Between group differences in CNS structures and neuropsychological tests**. Vertical bars represent group means, while error bars represent standard deviations. The horizontal lines over the vertical bars indicate significant between group differences after correction for multiple comparisons (*p* < 0.016). HC, healthy controls; aMCI, amnestic mild cognitive impairment; naMCI, non-amnestic mild cognitive impairment; CNS, central nervous system.

A significant difference was found in the volume of the hippocampus [*F*_(2, 61)_ = 9.2, *p* = 0.0002] and in the volume of the entorhinal cortex across study groups [*F*_(2, 61)_ = 4.3, *p* = 0.02]. The post hoc tests showed that the volumes are significantly decreased in the aMCI group relative to the controls and to the naMCI group, while the other two groups did not differ significantly (Table 3 and Figure 1) Among the covariates, gender had a significant effect on the volume of the hippocampus, male subjects had a significantly decreased hippocampus size relative to females [*F*_(2, 61)_ = 7.9, *p* = 0.007].

**Table 3:** **Differences of the CNS structures and the neuropsychological test results between study groups**.

**Structure**	**controls (*n* = 24)**	**naMCI (*n* = 18)**	**aMCI (*n* = 20)**	**Cohen's d**	**Cohen's d**	**Cohen's d**
	**Mean (SD)**	**Mean (SD)**	**Mean (SD)**	**(HC vs. aMCI)**	**(HC vs. naMCI)**	**(naMCI vs. aMCI)**
**CORTICAL THICKNESS**^a^						
Entorhinal cortex	3.485 (0.236)	3.318 (0.255)	2.803 (0.640)	1.6^*^	0.7	1.2^*^
Fusiform gyrus	2.619 (0.158)	2.538 (0.169)	2.376 (0.252)	1.2^*^	0.5	0.8^*^
Isthmus of cingulate gyrus	2.287 (0.169)	2.178 (0.137)	2.103 (0.202)	1^*^	0.7	0.4
Parahippocampal gyrus	2.678 (0.317)	2.593 (0.331)	2.499 (0.438)	0.5	0.3	0.2
Posterior cingulate	2.380 (0.103)	2.385 (0.126)	2.332 (0.170)	0.4	0	0.4
Precuneus	2.210 (0.092)	2.101 (0.113)	2.029 (0.155)	1.5^*^	1.1^*^	0.5
**VOLUMES**^b^						
Entorhinal cortex	0.328 (0.061)	0.345 (0.054)	0.285 (0.079)	0.6	−0.3	0.9^*^
Fusiform gyrus	1.701 (0.200)	1.631 (0.216)	1.556 (0.191)	0.7	0.3	0.4
Isthmus of cingulate	0.456 (0.046)	0.468 (0.043)	0.445 (0.051)	0.2	−0.3	0.5
Parahippocampal gyrus	0.383 (0.044)	0.394 (0.056)	0.367 (0.093)	0.2	−0.2	0.4
Posterior cingulate	0.578 (0.047)	0.604 (0.058)	0.586 (0.054)	−0.2	−0.5	0.3
Precuneus	1.654 (0.104)	1.665 (0.143)	1.621 (0.154)	0.3	−0.1	0.3
Hippocampus	0.780 (0.044)	0.743 (0.094)	0.637 (0.156)	1.4^*^	0.5	0.8^*^
Amygdala	0.310 (0.024)	0.286 (0.035)	0.268 (0.069)	0.9^*^	0.8	0.3
**NEUROPSYCHOLOGICAL TEST**						
ACE total score^c^	94.000 (3.297)	89.111 (5.144)	82.211 (7.391)	2.2^*^	1.2	1.1^*^
ACE anterograde memory^d^	26.000 (1.769)	24.556 (2.640)	20.842 (4.285)	1.7^*^	0.7	1.1^*^
ACE category fluency^d^	6.542 (0.658)	5.889 (1.323)	3.947 (1.580)	2.3^*^	0.7	1.3^*^
ACE letter fluency^d^	5.625 (1.096)	4.056 (1.830)	4.579 (1.427)	0.8	1.1^*^	−0.3
ACE language^d^	27.625 (0.711)	27.444 (1.338)	26.050 (6.211)	0.5	0.2	0.4
MMSE^e^	28.500 (1.351)	28.278 (0.958)	27.474 (1.679)	0.7	0.2	0.6
ACE Retrograde memory^d^	3.708 (0.955)	3.222 (0.878)	2.421 (1.346)	1.1^*^	0.5	0.7
ACE Visuospatial function^d^	4.792 (0.509)	4.389 (1.145)	4.421 (0.838)	0.6	0.5	0
Trail Making Test part A (sec)	41.917 (22.645)	59.222 (18.057)	75.650 (52.109)	−0.9^*^	−0.9	−0.5
Trail Making Test part B (sec)	68.542 (31.121)	153.18 (78.212)	199.74 (113.09)	−2^*^	−2^*^	−0.5

*HC, healthy controls; CNS, central nervous system; SD, standard deviation; aMCI, amnestic mild cognitive impairment; naMCI, non-amnestic mild cognitive impairment; ACE, Addenbrooke's cognitive examination; MMSE, Mini Mental State Examination*.

a *Cortical thickness in mm*.

b *Cortical volume in mm^3^*.

c *The maximum score is 100*.

d Subtasks of the ACE

e *Maximum score is 30.The normative data can be found in the Table 2B*.

* *Significant between group difference after correction for multiple comparisons (p < 0.016). Level of significance was set to 0.05/3 = 0.016, where three stands for the number of the study groups compared*.

The average thickness of the entorhinal cortex [*F*_(2, 61)_ = 13.1, *p* < 0.0001], the fusiform gyrus [*F*_(2, 61)_ = 6.7, *p* = 0.002], the isthmus of cingulated gyrus [*F*_(2, 61)_ = 5.4, *p* = 0.007], and the precuneus [*F*_(2, 61)_ = 10.4, *p* = 0.0001] are also differed significantly across the study groups. According to the *post-hoc* tests, decreased cortical thickness were found in the aMCI relative to the controls in all four structures, while the thickness of the entorhinal cortex and the fusiform gyrus were also significantly decreased in the aMCI group relative to the naMCI group. The group means, the results of the post-hoc tests, and the between group differences in terms of Cohen's d are summarized in Table 3.

There was a significant difference across study groups in anterograde memory [*F*_(2, 60)_ = 12.9, *p* < 0.0001], retrograde memory [*F*_(2, 60)_ = 6.1, *p* = 0.004], categorical fluency [*F*_(2, 60)_ = 22.9, *p* < 0.0001), and letter fluency [*F*_(2, 60)_ = 4.9, *p* = 0.01]. In anterograde memory and categorical fluency both the control group and the naMCI group outperformed the aMCI group, while the former groups did not differ significantly (Table 3). In retrograde memory controls outperformed the aMCI group, but not the naMCI group. In letter fluency controls outperformed the naMCI group, but not the aMCI group, while the two MCI groups did not differ from each other (*p* > 0.05). Furthermore in categorical fluency female subjects performed better than males [*F*_(1, 60)_ = 9.8, *p* = 0.003], while age had no significant effect on the performance.

### Correlational analyses

In the entire sample strong correlation were found between the results of the neuropsychological tests and the volume and thickness of the temporal brain structures (Table 4).

**Table 4:** **Correlations between the CNS structures and the neuropsychological test results across the entire sample**.

**Structure**	RAVLT items 1-5 sum (*n* = 62) Pearson r (p-value^*^)	ACE^a^ Total Score (*n* = 61) Pearson r (p-value^*^)	Trail Making Test part B (*n* = 60) Pearson r (p-value^*^)	Stages Completed in the PAL^b^ test (*n* = 49) Spearman r (p-value^*^)
**CORTICAL THICKNESS (IN mm)**				
Entorhinal cortex	**0.60 (*p* ≤ 0.001)**	**0.58 (*p* ≤ 0.001)**	0.20 (*p* = 0.129)	**0.47 (*p* ≤ 0.001)**
Fusiform gyrus	**0.52 (*p* ≤ 0.001)**	**0.53 (*p* ≤ 0.001)**	0.29 (*p* = 0.026)	**0.37 (*p* = 0.008)**
Isthmus of cingulate gyrus	**0.40 (*p* = 0.001)**	**0.37 (*p* = 0.004)**	**0.34 (*p* = 0.008)**	0.23 (*p* = 0.109)
Parahippocampal gyrus	**0.32 (*p* = 0.010)**	0.28 (*p* = 0.027)	0.14 (*p* = 0.269)	0.24 (*p* = 0.098)
Posterior cingulate	0.30 (*p* = 0.020)	**0.32 (*p* = 0.012)**	0.24 (*p* = 0.070)	0.24 (*p* = 0.100)
Precuneus	**0.48 (*p* ≤ 0.001)**	**0.44 (*p* ≤ 0.001)**	**0.40 (*p* = 0.001)**	0.35 (*p* = 0.013)
**VOLUMES (IN mm^3^)**				
Entorhinal cortex	0.31 (*p* = 0.013)	**0.36 (*p* = 0.004)**	0.12 (*p* = 0.342)	0.19 (*p* = 0.194)
Fusiform gyrus	0.28 (*p* = 0.030)	**0.37 (*p* = 0.004)**	0.29 (*p* = 0.027)	0.34 (*p* = 0.015)
Isthmus of cingulate	0.01 (*p* = 0.955)	0.04 (*p* = 0.752)	0.16 (*p* = 0.234)	0.09 (*p* = 0.543)
Parahippocampal gyrus	0.22 (*p* = 0.082)	0.25 (*p* = 0.053)	0.01 (*p* = 0.959)	0.14 (*p* = 0.353)
Posterior cingulate	0.13 (*p* = 0.308)	0.18 (*p* = 0.168)	0.30 (*p* = 0.020)	0.19 (*p* = 0.182)
Precuneus	0.02 (*p* = 0.890)	0.07 (*p* = 0.583)	0.24 (*p* = 0.065)	0.14 (*p* = 0.320)
Hippocampus	**0.50 (*p* ≤ 0.001)**	**0.51 (*p* ≤ 0.001)**	0.15 (*p* = 0.238)	**0.43 (*p* = 0.002)**
Amygdala	**0.40 (*p* = 0.001)**	**0.45 (*p* ≤ 0.001)**	0.06 (*p* = 0.623)	**0.51 (*p* ≤ 0.001)**

a *ACE = Addenbrooke's cognitive examination (maximum score is 100)*.

b *PAL test = Paired Associates Learning test*.

* *Significant between group differences after correction for multiple comparisons (p < 0.0125) are bolded. Level of significance was set to 0.05/4 = 0.0125, where four stands for the number of the neuropsychological tests analyzed*.

**Figure 2:**
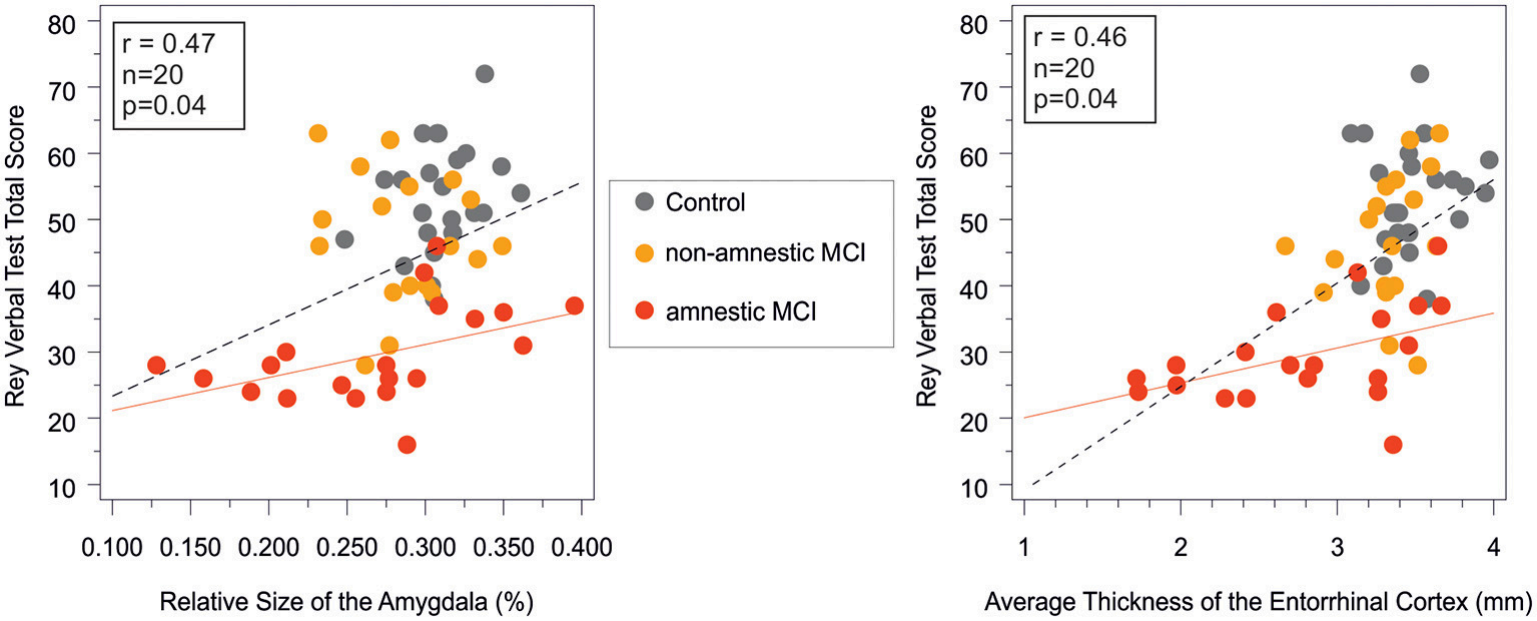
**Correlation between short term memory performance as indexed by the Rey verbal learning test and the amygdala volume and the entorhinal cortex thickness in the study groups**. Pearson correlations were significant (*p* < 0.05) in the amnestic MCI group and in the entire sample, while non-significant in the control and in the non-amnestic MCI groups. Rey verbal test total score on the vertical axes equals the sum of words in the first five trials. red line, regression line in the aMCI group; dashed line, regression line in the entire sample; MCI, mild cognitive impairment; r and p, Pearson correlation coefficient adjusted for age and corresponding level of significance in the aMCI group.

In the aMCI group the result of the Rey Verbal Learning Test showed a significant positive correlation with the size of the amygdala (*r* = 0.47, *n* = 20, *p* = 0.03), and the thickness of the entorhinal cortex (*r* = 0.46, *n* = 20, *p* = 0.04; Figure 2). Subjects with decreased amygdala volumes and decreased entorhinal thickness showed poorer performance on the memory test.

Furthermore subjects with decreased entorhinal volumes (*r* = 0.49, *n* = 19, *p* = 0.03), and decreased fusiform gyrus volumes (*r* = 0.48, *n* = 19, *p* = 0.04) and fusiform cortical thickness (*r* = 0.53, *n* = 19, *p* = 0.02) performed weaker in the retrograde memory subtest of the ACE.

## Discussion

Amnestic and non-amnestic MCI were assigned as potential prodromes to different types of NCDs. Subjects with amnestic mild cognitive impairment are assumed to have a higher risk of converting to Alzheimer's dementia, while subjects with non-amnestic mild cognitive impairment are said to have a higher risk of converting to non-Alzheimer's dementia (Petersen et al., 1999; Killiany et al., 2000; Petersen and Negash, 2008).

The structural differences of the brain were examined by MRI in the MCI subgroups and healthy control group, while the differences in cognitive performance were investigated by neuropsychological tests. According to MRI results the thickness of the entorhinal cortex, the fusiform gyrus, the isthmus of cingular gyrus, and the precuneus, and the volume of the amygdala and the hippocampus were decreased in aMCI relative to healthy controls. These findings are in line with previous studies reporting that healthy subjects, patients with MCI or Alzheimer's disease can be differentiated from each other based on the volumes of the temporal lobe structures such as the amygdala or the hippocampus (Desikan et al., 2009; McEvoy et al., 2009).

The volumes of the hippocampus and the entorhinal cortex, and the thickness of the entorhinal cortex and the fusiform gyrus are significantly decreased in the aMCI relative to the naMCI group. The largest between group difference was detected in the volume and thickness of the entorhinal cortex (0.9 and 1.2 SD, respectively), which is in line with the fact that atrophy in AD starts in this region. These results show that there are early structural differences between the subtypes of MCI. Furthermore the pattern of these structural findings are fit to the pathology of Alzheimer's disease (Braak and Braak, 1991), which underline those previous results that aMCI progress to Alzheimer disease with a higher frequency relative to naMCI (Killiany et al., 2000; Petersen and Negash, 2008).

Comparing the control and the naMCI group a significant difference was only found in the thickness of the precuneus which was decreased in the naMCI group. Interestingly a recent study found white matter lesions in patients with naMCI also in the precuneus (O'Dwyer et al., 2011), which is an important hub sustaining information transfer between the parahippocampal gyrus and the prefrontal cortex (Vincent et al., 2006) and plays an important role in memory process and visual imaginary.

Among the neuropsychological tests, the MMSE did not differ across study groups, which is a likely consequence of the exclusion of patients with dementia. In the ACE total, ACE anterograde memory, and ACE category fluency subtests the aMCI group performed worse relative to the naMCI group and the healthy control group. In the retrograde memory test of the ACE and in the Trail Making A and B tests the aMCI group only performed worse than the healthy controls, while no difference was detected between the two MCI subgroups. The decreased performance on the memory tasks (RAVLT and ACE retrograde memory task) in the aMCI group correlated with the increased involvement of temporal lobe structures such as the entorhinal cortex, and the amygdala (Figure 2), which further confirms our results on between group differences.

In the letter fluency task the naMCI group performed worse than the healthy group, while the aMCI did not differ from the other groups. Trail making B test is an index of visual attention and task switching. The decreased performance of both MCI groups relative to controls (Table 3) is likely the result of frontal involvement. Both category and letter fluency tasks rely strongly on the functioning of the frontal lobe, including executive processes that require subjects to organize retrieval, monitor responses previously recalled, initiate verbal responses and inhibit responses that do not fit within the criteria (Henry et al., 2004). “Both measures also access semantic memory stores, a function of the temporal lobe; although letter fluency appears to tap this ability linked to temporal lobe to a lesser extent than category fluency” (Lezak et al., 2004). Lesion and functional brain imaging studies also support the involvement of the temporal and frontal lobes in fluency ability. Previous fMRI studies showed that Letter fluency is associated with increased activation in the frontal lobes, while both the frontal and temporal lobes are active during category fluency (Birn et al., 2010). This corresponds well with our findings that aMCI differs significantly according to category fluency in comparison with healthy and naMCI group, while there is no significant difference between the two subgroups in the letter fluency result.

Strong correlations were found between verbal and visual memory functions as indexed by the RAVLT and the PAL tests, and the volumes and cortical thickness of the temporal structures such as the entorrhinal cortex (Table 4) in the entire sample and also in the aMCI group (Figure 2). These findings cross validate the usefulness of these neuropsychological and MRI markers in the early diagnosis of pathological cognitive decline and in the monitoring of disease progression.

### Limitations

A limitation of the study is that the majority of the subjects in the aMCI group were multi-domain type, since their performance in executive functions as assessed by the trail making test B and the ACE were under the normal range. This limitation taken together with the fact that naMCI is a more heterogeneous entity relative to aMCI may explain why the current study was not able to find CNS structures with decreased size in the naMCI group relative to the aMCI group.

## Conclusions

The naming of aMCI and naMCI are not just theoretical but these subtypes are different entities both from a neuropsychological and from a brain structural viewpoint. The development of specific MRI and neuropsychological criteria for the different subtypes of mild cognitive impairment will then make it possible to assess the determinants and the prevalence of the MCI subtypes.

The assignment of MCI subtypes will be useful to improve the prediction of dementia type and the risk of conversion to dementia. Furthermore the assignment of MCI subtypes may provide a better approach to testing the efficacy of therapeutic options in preventing the conversion to neurocognitive disorders. Based on our results MRI can be a useful tool to the more precise separation between MCI subtypes.

## Author contributions

GC designed the study, wrote the protocol, undertook the statistical analysis, created the figures, and wrote the first draft of the manuscript ES participated in the execution of measurements, managed the literature searches and analyses, and wrote the first draft of the introduction and the conclusion. ÁS contributed to the finalization of all sections of the manuscript. ZF, AH participated in the execution of measurements, and contributed to the writing of the methods section. ÉC, PS, ZH, GR gave supervision for the experiments during the study including the writing of the manuscript. All authors contributed to and have approved the final manuscript.

## Funding

The study was supported by the “Ambient Assisted Living Joint Programme (AAL)—Call 2” grant (Project Identifier: AAL_08-1-2011-0005 M3W), (http://www.aal-europe.eu).

### Conflict of interest statement

The authors declare that the research was conducted in the absence of any commercial or financial relationships that could be construed as a potential conflict of interest.
